# Targeted sequencing analysis of *PPARG* identifies a risk variant associated with obstructive sleep apnea in Chinese Han subjects

**DOI:** 10.1007/s11325-019-01855-x

**Published:** 2019-05-02

**Authors:** Xiaolu Jiao, Song Yang, Yunyun Yang, Juan Li, Haili Sun, Ming Zhang, Yunxiao Yang, Yanwen Qin

**Affiliations:** 1 Key Laboratory of Remodeling-related Cardiovascular Diseases, Beijing Anzhen Hospital, Beijing Institute of Heart, Lung and Blood Vessel Diseases, Capital Medical University, No. 2 Anzhen Road, Chaoyang District, Beijing, 100029 China; 2 Key Laboratory of Upper Airway Dysfunction-related Cardiovascular Diseases, Beijing Anzhen Hospital, Beijing Institute of Heart, Lung and Blood Vessel Diseases, Capital Medical University, Beijing, 100029 China; 3 Otolaryngological Department of Beijing Anzhen Hospital, Capital Medical University, Beijing, 100029 China; 4 Department of Cardiology, Beijing Anzhen Hospital, Capital Medical University, Beijing, 100029 China

**Keywords:** Obstructive sleep apnea, Peroxisome proliferator-activated receptor gamma, Single nucleotide polymorphisms, Targeted sequencing

## Abstract

**Purpose:**

Obstructive sleep apnea (OSA) is a common disorder characterized by recurrent episodes of partial or complete upper airway obstruction. OSA susceptibility is associated with multiple genetic, environmental, and developmental factors. The *PPARG* rs1801282 (G/C) polymorphism has been associated with OSA in obese Indian subjects, whereas no such association has been reported in Chinese Han subjects. Potential associations between other *PPARG* variants and OSA have not been investigated in Chinese Han populations. The aim of this study was to identify genetic variants of *PPARG* in unrelated Chinese Han patients with OSA and to investigate potential associations between these variants and OSA.

**Methods:**

We performed a cross-sectional study of 233 individuals with OSA and 93 control individuals. A portable diagnostic device was used to diagnose OSA. Targeted sequencing was conducted to identify *PPARG* variants. Associations between *PPARG* variants and OSA were analyzed using multivariate regression.

**Results:**

Three *PPARG* single-nucleotide polymorphisms were identified and the genotype frequencies of the rs1801282 polymorphism differed significantly. Subjects with the *PPARG* rs1801282 CG genotype had decreased risk of having OSA compared with subjects with the CC genotype after adjusting for confounding effects.

**Conclusions:**

We identified a variant of *PPARG* associated with the occurrence of OSA in Chinese Han populations.

**Electronic supplementary material:**

The online version of this article (10.1007/s11325-019-01855-x) contains supplementary material, which is available to authorized users.

## Introduction

Obstructive sleep apnea (OSA) is a relatively common sleep disorder characterized by recurrent episodes of partial or complete collapse of the upper airway during sleep, leading to loud snoring, sleep fragmentation, daytime sleepiness, and chronic episodes of intermittent hypoxia [1]. It has been estimated that 34% of men and 17% of women are affected by OSA [2, 3]. Patients with OSA are at high risk of hypertension, diabetes, cardiovascular diseases, stroke, and other disorders [1, 4]. Previous studies have reported multiple risk factors for OSA [5–9], including obesity, male sex, micrognathia, menopause, fluid retention, adenotonsillar hypertrophy, and smoking. However, the pathogenesis of OSA is not fully understood.

OSA clusters within families [5] and having a first-degree relative with OSA increases the risk of OSA by more than 1.5-fold. The apnea–hypopnea index (AHI) is the most commonly used metric for OSA, and approximately 35% to 40% of variation in AHI can be explained by genetic factors [6, 10–13]. Other than male sex, the primary risk factor for OSA is excessive weight gain and obesity is the strongest risk factor for the development of OSA, and at least 70% of OSA patients are obese [14]. Peroxisome proliferator-activated receptor gamma (PPARG) is a member of the nuclear receptor family that includes 48 human transcription factors [15]. PPARG has been linked to development of obesity [15–17] and may play an important role in the pathophysiological mechanisms underlying OSA. PPARG expression was downregulated in the adipose tissue of OSA patients relative to control individuals, as measured using qPCR [18].

The *PPARG* rs1801282 (G/C) polymorphism was reported to be associated with OSA in obese Indian subjects [19], whereas no association between rs1801282 (G/C) and OSA was observed in a Chinese Han population [20]. Potential associations between other *PPARG* variants and OSA have not been investigated in Chinese Han individuals.

In this study, we aimed to identify genetic variants of *PPARG* by targeted sequencing in unrelated Chinese Han subjects and to explore potential associations between OSA and the identified variants.

## Methods

### Subjects

This was a cross-sectional study. In total, 326 unrelated Chinese Han individuals aged ≥ 18 years were recruited, including 233 patients with OSA and 93 controls. The study flow chart is shown in Supplemental Data Fig. S1. Patients who were seen in the Otolaryngological Department at Beijing Anzhen Hospital between April 2017 and November 2017 were screened for OSA using the Berlin questionnaire, which contains three categories of questions related to risk of sleep apnea: snoring and cessation of breathing, daytime sleepiness, and obesity or hypertension. Subjects are considered to be at high risk if their scores are positive in two or more categories [21] and are otherwise considered at low risk for OSA. Patients with respiratory diseases requiring medication, congestive heart failure, cancer, acute infectious diseases, hepatic dysfunction, abnormal renal function, or current pregnancies were excluded [22, 23]. Patients with physician-diagnosed narcolepsy, idiopathic hypersomnolence, chronic insomnia, restless legs syndrome, or any other sleep disorder were also excluded [22]. The diagnosis of narcolepsy, idiopathic hypersomnolence, chronic insomnia, restless legs syndrome, or any other sleep disorder were depended on medical histories in this study. We also excluded patients who used hypnotics, anxiolytics, sedating antidepressants, anticonvulsants, sedating antihistamines, stimulants, or other medications likely to affect the function of the nervous system [22, 23]. Finally, we selected a total of 360 eligible subjects. Each eligible subject was scheduled for an overnight sleep study, which was conducted using a level II portable diagnostic device (SOMNOscreen; SOMNOmedics GmbH, Randersacker, Germany) approved by the US Food and Drug Administration. The SOMNOscreen device assesses airflow by means of a nasal cannula pressure transducer and an oronasal thermal sensor, thoracic and abdominal movement using inductance plethysmography, and oxygen saturation by means of pulse oximetry with a fingertip sensor. Patients who were at low risk based on the Berlin questionnaire and had an AHI ≥ 5 based on the results of overnight sleep study, as well as patients who were at high risk and had an AHI < 15, were excluded. Individuals who were at low risk based on the Berlin questionnaire and had AHI < 5 were classified as controls, while individuals who were at high risk and had AHI ≥ 15 were classified as having OSA. Finally, a total of 326 subjects were included in this study, including 233 patients with OSA and 93 controls. All participants gave written informed consent before enrollment. The protocol was approved by the Medicine Ethics Committee of Beijing Anzhen Hospital (2017005) and adhered to the principles laid out in the Declaration of Helsinki. This study is registered in the Chinese Clinical Trial Registry (No. ChiCTR-ROC-17011027).

OSA was defined as having an AHI of ≥ 15 events per hour according to the American Academy of Sleep Medicine guidelines [24–26]. In this study, the quantitative outcomes were the AHI and the lowest oxygen saturation (SpO2) across the recording period [27]. The AHI was calculated as the number of apnea and hypopnea events recorded per hour, where apnea was defined as breathing cessation for > 10 s and hypopnea was defined as respiratory airflow reduced by 30% with a 4% decrease in SpO2 [24]. Epworth Sleepiness Scale (ESS) was used to identify daytime sleepiness.

Anthropometric determinations and blood extractions were performed on a single day as described previously [28]. Blood samples were taken after overnight fasting, and routine laboratory analyses were performed to measure fasting blood glucose, lipid profiles, and other blood chemistry markers. Weight and height were measured (subjects wore light indoor clothing and were barefoot) and the body mass index (BMI) (kg/m^2^) was calculated using the formula: weight (kilograms) divided by height (meters) squared. According to the recommendations of the Health Promotion Administration, obesity was defined as BMI ≥ 28 kg/m^2^ [29]. Blood pressure was measured at the right upper arm after a 5-min rest in a sitting position at least three times, and the mean value was used in the analyses. Nonsmokers were subjects who had never smoked or had stopped smoking for ≥ 1 year before enrollment in the study. All remaining subjects were classified as smokers. Drinkers were defined as subjects who consumed alcohol ≥3 days a week. The definitions of dyslipidemia, diabetes, and hypertension were based on subject medical histories.

### DNA template preparation and amplification

Genomic DNA was extracted from 200 μL of whole blood using a QIAamp DNA Mini Kit (Qiagen, Hilden, Germany) according to the manufacturer’s protocol. The multiplex PCR amplification strategy to amplify target DNA sequences was designed online (Ion Ampliseq™ Designer; http://www.ampliseq.com). Primer sequences are listed in Supplemental Data Table S1. After evaluating the primer design options, the design that provided the maximum coverage was chosen. The amplicons covered approximately 99.80% of the target sequence. Ten nanograms of DNA per sample was used for enrichment by multiplex PCR. Each DNA pool was amplified using an Ion Ampliseq™ Library Kit in conjunction with the Ion Ampliseq™ Custom Primer Pool according to the manufacturer’s protocols (Life Technologies, Darmstadt, Germany).

After each pool had undergone 17 PCR cycles, the PCR primers were removed with FuPa Reagent (Life Technologies) and the amplicons were ligated to sequencing adaptors containing short barcodes that enabled sample multiplexing using an Ion Xpress™ Barcode Adapters Kit (Life Technologies). After purification with AMPure XP beads (Beckman Coulter, Krefeld, Germany), the barcoded libraries were quantified with a Qubit® 2.0 Fluorimeter (Life Technologies) and the DNA concentration was normalized to a final concentration of 20 pmol/L using an Ion Library Equalizer™ Kit (Life Technologies). Equimolar amounts of barcoded libraries from 64 samples at a time were pooled. To clonally amplify the library DNA onto Ion Sphere Particles (Life Technologies), the library pool was subjected to emulsion PCR using an IT OneTouch Template kit on an IT OneTouch system (Life Technologies) following the manufacturer’s protocol.

### Targeted sequencing

The *PPARG* sequence was obtained using Ion Torrent semiconductor sequencing (Life Technologies, Carlsbad, CA, USA). Enriched Ion Sphere Particles that carried many copies of the same DNA fragment were sequenced on an Ion 318 Chip to sequence pooled libraries containing 64 samples at a time. Sequencing was performed using an Ion PGM Sequencing kit (Life Technologies) with the 400-bp single-end run configuration according to the manufacturer’s instructions. Details of the primers used are given in Supplemental Data Table S2.

### Computational analysis

Torrent Suite software version 4.4.2 (Life Technologies, Darmstadt, Germany) was used to align reads from the raw data (unmapped BAM files). The read alignments were filtered by Torrent Suite into mapped BAM files using the human reference genomic sequence (hg19) of *PPARG*. Variant calling was performed with the Torrent Variant Caller Plugin (minimum allele frequency 0.2; minimum quality 20; minimum coverage 20; and minimum coverage on either strand 3). Annotation of variants was performed using Ion Reporter software version 4.4 (Life Technologies, Darmstadt, Germany) for the variant call format files. The annotations included genomic and complementary DNA positions, genetic reference sequences, amino acid changes, and related information available from public databases such as the 1000 Genomes Project, the National Heart, Lung, and Blood Institute Grand Opportunity Exome Sequencing Project (ESP6500) (https://esp.gs.washington.edu/drupal/), the Single Nucleotide Polymorphism Database (dbSNP147) (National Center for Biotechnology Information, http://www.ncbi.nlm.nih.gov/SNP/), the Exome Aggregation Consortium (ExAC03) (http://exac.broadinstitute.org), ClinVar, DrugBank, Online Mendelian Inheritance in Man (OMIM), and the Human Gene Mutation Database (HGMD).

### Statistical analysis

Continuous variables were expressed as means ± standard deviations (for normally distributed data) or medians (interquartile ranges) (for asymmetrically distributed data) and categorical variables were expressed as numerals (percentages). Independent Student’s *t* tests (for normally distributed data) and Wilcoxon rank sum tests (for asymmetrically distributed data) were used to analyze differences in continuous variables. Chi-squared tests and Fisher’s exact tests were used to analyze categorical variables. Deviations of genotype frequencies from the assumption of Hardy–Weinberg equilibrium were assessed using a chi-square test. Associations between OSA and *PPARG* variants were analyzed by logistic regression analysis. Step-down Bonferroni correction was used to adjust for multiple comparisons in exploring the association between OSA and PPARG variants. All probability values were two-sided, and a *P* value <0.05 was considered statistically significant. All analyses were performed with R (http://www.R-project.org) and Empower Stats software (www.empowerstats.com; X&Y Solutions, Inc., Boston, MA, USA).

## Results

### Baseline clinical characteristics of the study population

The clinical characteristics of the 233 individuals with OSA and 93 controls who participated in this study are shown in Table 1. There were no significant differences in age (*P* = 0.122), systolic blood pressure (*P* = 0.091), total cholesterol (*P* = 0.585), low-density lipoprotein cholesterol (*P* = 0.636), and fasting blood glucose (FBG; *P* = 0.201) between the two groups. Subjects with OSA had significantly higher BMIs and ESS (*P* = 0.018) than the non-OSA subjects (*P* < 0.001). Individuals with OSA also had higher diastolic blood pressure (*P* = 0.032), triglycerides (TGs) (*P* < 0.001), AHI (*P* < 0.001), and lower SpO2 (*P* < 0.001) than control individuals. A greater proportion of individuals with OSA were male, obese, smokers, and had hypertension or dyslipidemia compared with the non-OSA group.

**Table 1:** Anthropometric and biochemical characteristics of the subjects included in the study

	OSAHS *N* = 233	Non-OSAHS *N* = 93	*P* value
Age (years)	55.29 ± 10.47	52.82 ± 13.79	0.122
Male (n,%)	200 (85.84%)	66 (70.97%)	0.002*
BMI (kg/m^2^)	27.52 ± 3.50	24.02 ± 3.58	< 0.001**
Obesity (n,%)	99 (42.49%)	15 (16.13%)	< 0.001**
Smoker (n,%)	102 (43.78%)	27 (29.03%)	0.009*
Drinker (n,%)	98 (42.06%)	35 (37.63%)	0.276
Hypertension (yes, N n,%)	59 (25.32%)	12 (12.90%)	0.009*
Diabetes (yes, N n,%)	16 (6.87%)	5 (5.38%)	0.340
Dyslipidemia (yes, N n,%)	175 (75.11%)	54 (58.06%)	0.002*
SBP (mmHg)	126.45 ± 18.49	122.66 ± 17.56	0.091
DBP (mmHg)	77.31 ± 12.38	74.10 ± 11.66	0.032*
FBG (mmol/L)	4.67 ± 1.40	4.36 ± 2.03	0.201
TG (mmol/L)	1.59 (1.12–2.18)	1.27 (0.94–1.62)	< 0.001**
TC (mmol/L)	4.31 ± 1.08	4.38 ± 1.06	0.585
LDL-C (mmol/L)	2.55 ± 0.95	2.60 ± 0.83	0.636
HDL-C (mmol/L)	1.05 ± 0.27	1.14 ± 0.28	0.004*
AHI (times/h)	28.60 (21.00–42.80)	2.60 (1.65–3.50)	< 0.001**
Lowest SpO2 (%)	80.88 ± 11.08	90.42 ± 2.67	< 0.001**
ESS	6.00 (2.00–10.00)	*3.00 (1.00–6.00)*	0.018*

Results are expressed as mean ± standard deviation, median (interquartile range), or *n* (%). Differences between groups were analyzed by independent Student *t* test, Fisher’s exact test, χ^2^ text, or Wilcoxon test

*OSA* obstructive sleep apnea, *BMI* body mass index, *SBP* systolic blood pressure, *DBP* diastolic blood pressure, FPG fasting plasma glucose, *TG* triglycerides, *TC* total cholesterol, *LDL-C* low-density lipoprotein cholesterol, *HDL-C* high-density lipoprotein cholesterol, *AHI* apnea–hypopnea index, *ESS* Epworth Sleepiness Scale

*P < 0.05; **P < 0.001

### PPARG sequencing and SNP detection

We used a targeted next-generation sequencing approach to analyze *PPARG* gene sequences in 233 individuals with OSA and 93 controls. A total of 17 variants were identified in the *PPARG* sequence but only seven had a rs number. The details of the variants are shown in Supplemental Data Table S3. Among the seven *PPARG* variants, three were not represented in the 1000 Genomes Project database and were present in only one study subject. Three variants (rs1801282, rs3856806, and rs13073869) were single-nucleotide polymorphisms (SNPs) according to the 1000 Genomes Project database, whose minimum allele frequency is ≥ 0.01. As shown in Supplemental Data Table S3, we also identified three rare non-synonymous variants (p.Y95C, p.V403A, and p.H30Y) which may be deleterious according to the SIFT and PolyPhen-2 databases. However, further study is required to determine the functions of these rare variants.

### Association of PPARG variants with the risk of having OSA

Three SNPs in the *PPARG* sequence were identified in this study. Their allele frequency distributions are shown in Supplemental Data Table S4. All three SNPs were in Hardy–Weinberg equilibrium. The genotype frequencies at the rs1801282 SNP differed significantly (*P* < 0.05, Table 2) between the OSA and control group. The frequency of the *PPARG* rs1801282 G allele was significantly lower in subjects with OSA (4.72%) compared with subjects without OSA (10.22%; *P*^a^ = 0.027, Table 2). For the other two SNPS (rs3856806 and rs13073869), no significant differences were observed between the genotypes and allele distributions of the OSA and control groups. We also found that subjects with the *PPARG* rs1801282 CG genotype had decreased risk of having OSA compared with subjects with the CC genotype after adjusting for age, sex, BMI, smoking, alcohol consumption, TGs, high-density lipoprotein cholesterol (HDL-C), FBG, and ESS (OR = 0.318, 95% CI 0.136–0.746; *P*^a^ = 0.008) (Table 3). No significant differences between the two groups were detected for the other two polymorphisms after adjustment for confounders.

**Table 2:** Genotype frequencies in OSA and non-OSA subjects

SNPs	Genotype	OSA group (N/%) *N* = 233	Non-OSA group (N/%) s*N* = 93	χ2	*P*^a^
rs1801282	CC	211 (90.56%)	74 (79.57%)	7.299	0.021*
	CG	22 (9.44%)	19 (20.43%)		
	C	444 (95.28%)	167 (89.78%)	6.089	0.027*
	G	22 (4.72%)	19 (10.22%)		
rs3856806	CC	147 (63.09%)	51 (54.84%)	2.173	0.337
	CT	80 (34.33%)	38 (40.86%)		
	TT	6 (2.58%)	4 (4.30%)		
	C	374 (80.26%)	140 (75.27%)	1.983	0.194
	T	92 (19,74%)	46 (24.73%)		
rs13073869	GG	96 (41.20%)	41 (44.09%)	5.810	0.111
	GA	116 (49.79%%)	36 (38.71%)		
	AA	21 (9.01%)	16 (17.20%)		
	G	308 (66.09%)	118 (63.44%)	0.413	0.290
	A	158 (33.91%)	68 (36.56%)		

Results are expressed as *n* (%). Differences between groups were analyzed by χ^2^ text or Fisher’s exact

*OSA* obstructive sleep apnea, *SNPs* single nucleotide polymorphisms, *P*^a^*P* value was adjusted by step-down Bonferroni

*P < 0.05

**Table 3:** Multivariate logistic regression analyses of three SNPs in PPARG gene with the risk of OSA in all subjects

SNPs	Genotype	unadjusted		Model 1		Model 2	
		OR (95CI)	*P*^a^	OR (95CI)	*P*^a^	OR (95CI)	*P*^a^
rs1801282	CC (*N* = 285)	1		1		1	
	CG (*N* = 41)	0.406 (0.208–0.792)	0.040*	0.278 (0.126–0.612)	0.005*	0.318 (0.136–0.746)	0.008*
rs3856806	CC (*N* = 198)	1		1		1	
	CT (*N* = 118)	0.730 (0.443–1.205)	0.657	1.393 (0.731–2.654)	0.626	0.898 (0.479–1.683)	0.898
	TT (*N* = 10)	0.520 (0.141–1.918)	0.326	0.635 (0.234–1.726)	0.373	0.684 (0.125–3.737)	0.684
rs13073869	GG (*N* = 137)	1		1		1	
	GA (*N* = 152)	1.376 (0.816–2.322)	0.462	1.378 (0.752–2.523)	0.897	1.643 (0.856–3.153)	0.136
	AA (*N* = 37)	0.561 (0.266–1.182)	0.512	0.552 (0.230–1.324)	0.732	0.654 (0.258–1.657)	0.371

Model 1: adjusted for age, sex, BMI, smoker and drinker. Model 2: adjusted for model 1 + TG, HDL-C, FBG and ESS

*SNPs* single nucleotide polymorphisms, *PPARG* peroxisome proliferator-activated receptor gamma, *OSA* obstructive sleep apnea, *BMI* body mass index, *TG* triglycerides, *HDL-C* high-density lipoprotein cholesterol, *FPG* fasting plasma glucose, *ESS* Epworth Sleepiness Scale, *P*^a^*P* value was adjusted by step-down Bonferroni

*P < 0.05

Obesity is the most important risk factor for OSA and PPARG is related to obesity. To test if *PPARG* genotype was associated with OSA independent of obesity, obesity was stratified (Table 4). We found that obese subjects with the *PPARG* rs1801282 CG genotype had decreased risk of having OSA compared with subjects with the CC genotype after adjusting for confounding factors (OR = 0.166, 95%CI 0.030–0.901, *P* = 0.037). Non-obese subjects with the *PPARG* rs1801282 CG genotype also had decreased risk of having OSA compared with subjects with the CC genotype after adjusting for confounding factors (OR = 0.345, 95%CI 0.124–0.959, *P* = 0.039). The risk of rs1801282 to obesity was also analyzed in this study; we found that after adjusting for confounding factors, there were no differences between subjects with *PPARG* rs1801282 CC genotype and CG genotype about risk of obesity in all subjects, OSA subjects, and non-OSA subjects in this study (Supplemental Data Table S5).

**Table 4:** Multivariate logistic regression analyses of rs1801282 with the risk of OSA in stratified analysis according to obesity

	Genotype	Unadjusted		Model 1		Model 2	
		OR (95CI)	*P* value	OR (95CI)	*P* value	OR (95CI)	*P* value
Obesity *N* = 114	CC	1		1		1	
	CG	0.188 (0.056–0.628)	0.007*	0.150 (0.039–0.580)	0.006*	0.166 (0.030–0.901)	0.037*
Non-obesity *N* = 212	CC	1		1		1	
	CG	0.447 (0.190–1.054)	0.066	0.368 (0.139–0.977)	0.045*	0.345 (0.124–0.959)	0.041*

Model 1: adjusted for age, sex, BMI, smoker and drinker. Model 2: adjusted for model 1 + TG, HDL-C, FBG and ESS

*OSA* obstructive sleep apnea, *BMI* body mass index, *TG* triglycerides, *HDL-C* high-density lipoprotein cholesterol, *FPG* fasting plasma glucose, *ESS* Epworth Sleepiness Scale

*P < 0.05

## Discussion

The relationship between *PPARG* polymorphisms and OSA was investigated in individuals with and without OSA. Our results showed that the *PPARG* rs1801282 polymorphism was independently associated with OSA.

OSA is a complex disease that is influenced by a combination of genetic [12, 30] and environmental factors. The molecular mechanisms underlying OSA are not well understood. From a pathological viewpoint, several factors can cause OSA, including sympathetic nervous system activity, obesity, upper airway dilator muscle dysfunction, craniofacial abnormalities, heightened chemosensitivity, and a low arousal threshold [9, 31]. These pathological processes are also influenced by genes.

PPARG is known to affect obesity, adipose and muscle tissue metabolism, and craniofacial abnormalities thereby causing OSA. Obesity is the strongest risk factor for the development of OSA [14], and PPARG plays an important role in the development of obesity [15, 16]. PPARG regulates lipid and glucose metabolism, and downregulation of PPARG has been shown to have anti-obesity effects [32]. PPARG activation decreased free fatty acid levels and increased lipid storage in adipose tissue [33]. It was reported that craniofacial abnormalities and dysfunction of adipose and muscle tissue metabolism may cause upper airway obstruction, which is another risk factor for OSA. Roles of *PPARG* in regulating human adipocyte differentiation and adipogenesis have been identified [17]. Chang et al. [34] found that the progression of myogenesis was regulated by the level of intracellular PPARG. *PPARG* polymorphisms may be involved in the growth hormone/STAT5B pathway [35], and mice that were deficient in members of this pathway developed craniofacial abnormalities [30]. The growth hormone/STAT5B pathway also plays an important role in regulating energy metabolism in adipose and muscle tissue, suggesting that PPARG may cause craniofacial abnormalities and dysfunction of adipose and muscle tissue metabolism through this pathway [36, 37]. In addition, the rs1801282 SNP is located in an exon and is a missense variant that affects the amino acid sequence of the PPARG protein (Pro12Ala). Together, these findings indicated that variants in *PPARG* may affect the development of OSA, although the exact mechanism remains unclear.

We found that the frequency of the *PPARG* G allele was significantly lower in subjects with OSA compared with subjects without OSA. This association was present both in obese and non-obese individuals with OSA. Bharat et al. previously found that the frequency *of the PPARG* G allele was significantly higher in subjects with OSA when compared to obese control [19]. It was reported that allele subtypes differed in lean, overweight, and obese subjects [38]. However, the results of our study were inconsistent with those of Bharat et al. in obese subjects. All of the individuals included in our study were Chinese Han while the individuals studied by Bharat et al. were Asian Indians. This further illustrates the genetic differences at the *PPARG* locus between different ethnic groups.

We also found that rs1801282 was associated with OSA, whereas previously, Guan et al. [20] found no association between rs1801282 and OSA in a Chinese Han population. We found that the Guan et al.’s study was designed as a case-control and our study was a cross-sectional study, which may be the reasons that there were differences between Guan et al.’s study and our study. We hypothesized that genes linked with metabolic disorders may be relevant to the genetics of OSA because of the prominence of metabolic disorders in the syndrome’s phenotype. The *PPARG* rs1801282 polymorphism has attracted much attention because of its correlation with various metabolic conditions including obesity, diabetes, and dyslipidemia. In this study, we confirmed that the *PPARG* rs1801282 polymorphism was associated with OSA.

Previous studies showed that age was an independent risk factor for OSA [9] and that most individuals with OSA were male [9]. In our study, there were more male individuals in the OSA group than in the non-OSA group, so to avoid confounding effects, we adjusted for age and sex. Obesity plays an important role in OSA. *PPARG* rs1801282 polymorphisms were associated with decreased BMI and may slightly protect against childhood obesity [39]. BMI is associated with PPARG and adipogenesis is affected by PPARG [19], a known regulator of lipid and glucose metabolism. We found that individuals with OSA had higher BMI and TGs but lower HDL-C than non-OSA subjects. To avoid confounding effects, we also adjusted for BMI, TGs, HDL-C, and FBG.

Care was taken to avoid bias in this study. Genomic DNA extraction and targeted sequencing were performed according to the manufacturer’s instructions by a trained experimentalist who was unaware of the subjects’ clinical status. In the statistical analysis, adjustments were made for the confounding effects of risk factors for OSA and *PPARG*. Finally, the cross-sectional design of the study and consecutive recruitment of subjects reduced the effects of outcome-selection bias.

Despite this, our study had some limitations. First, the sample size was small and larger studies are needed to confirm our results. Second, the participants may not be entirely representative of the general Han Chinese population. Potential false-positive results may still be possible after multiple corrections. Prospective cohort studies are needed to confirm the variants and associations identified in our study. Third, the controls of this study were also from Otolaryngological Department but not from the population. Finally, the exact mechanisms of the identified *PPARG* variants is not fully understood and requires further functional studies.

In conclusion, we identified a *PPARG* variant in patients with OSA. The variant was predicted to be associated with the occurrence of OSA in Chinese Han subjects. Individuals with the *PPARG* rs1801282 CG genotype were at lower risk of having OSA than individuals with the CC genotype after adjusting for confounding effects. Genetic analysis may be helpful for individualized typing of patients with OSA and could contribute to personalized diagnosis and treatment.

## Electronic supplementary material

ESM 1 (DOCX 156 kb)

## Abbreviations

OSA: Obstructive sleep apnea
PPARG: Peroxisome proliferator-activated receptor gamma
SNP: Single nucleotide polymorphism
AHI: Apnea–hypopnea index
BMI: Body mass index
TG: Triglyceride
HDL-C: High-density lipoprotein cholesterol
FBG: Fasting blood glucose
