# Supplementary material for: Targeted sequencing analysis of PPARG identifies a risk variant associated with obstructive sleep apnea in Chinese Han subjects
Source: Sleep Breath. 2019 May 2;24(1):167–74. doi: 10.1007/s11325-019-01855-x (PMC7127989; doi:10.1007/s11325-019-01855-x)
Supplement: Supplementary file 1 — (DOCX 156 kb) [file 11325_2019_1855_MOESM1_ESM.docx]

**Table S1** Primers of PPARG gene

| Ion_AmpliSeq_Fwd_Primer* | Ion_AmpliSeq_Rev_Primer* | Name | Amplicon_Start | Insert_Start | Insert_Stop | Amplicon_Stop |
| --- | --- | --- | --- | --- | --- | --- |
| CATTTCTCTTTCTGAAACTCTGTGAGATTG | CAGGGTTACTGAGAGATGAGTCCA | PPARG | 12421124 | 12421154 | 12421473 | 12421497 |
| AATCGTGTCAAGAACCTGCCTT | TCTAAATTCTGGCATTTGGTGGAAAAAC | PPARG | 12433997 | 12434019 | 12434341 | 12434369 |
| AGCAAAGCAAGTTTACATAAACAGTTTTCT | GTTCAAGTCAAGATTTACAAAACCAGGA | PPARG | 12458102 | 12458132 | 12458375 | 12458403 |
| GGGAAAGCAAACATTCAGAATGAAATCC | GTCCAACTGACTGGAAAATTATATAGCAGA | PPARG | 12353734 | 12353762 | 12354078 | 12354108 |
| AAACGACACCAGGTAGCCTG | GGGCACCCGTACTCTGA | PPARG | 12329163 | 12329183 | 12329520 | 12329537 |
| TCTGATTCCATCCTTAGTTCTTACCCA | CTTCTTGATCACCTGCAGTAGCT | PPARG | 12475210 | 12475237 | 12475561 | 12475584 |
| GCTTGCCCTGTTGCCTTTTTAG | AGAGCAAGATACTCTTCCATGAAGAAAAA | PPARG | 12422752 | 12422774 | 12423083 | 12423112 |
| GGAGAGCACAGTGTGTGTTCAG | CGCAGTAAACCATTTACTCAAAGAAACAAA | PPARG | 12447311 | 12447333 | 12447633 | 12447663 |
| CTGTGCAGGAGATCACAGAGTAT | ACCCTCTTTCATAGAAGATCAAAAGACAAA | PPARG | 12458342 | 12458365 | 12458659 | 12458689 |
| GGTAGAGGCAGCAGAGGTTAAC | CAACACATTCCAACAAAATGCAATGA | PPARG | 12330398 | 12330420 | 12330649 | 12330675 |
| GCCAATTCAAGCCCAGTCCTTT | GTATTAAAACAAACACAACCTGGAAGACAA | PPARG | 12392902 | 12392924 | 12393245 | 12393275 |
| GACAGACCTCAGACAGATTGTCAC | GGGAAACACACAAGACTCAGTCT | PPARG | 12475527 | 12475551 | 12475860 | 12475883 |

**Table S2** AmpliSeq™ amplicons and coverage details of obstructive sleep apnea-targeted sequencing assay

| Target_ID | Gene_Symbol | Chromosome | Chr_Start | Chr_End | Num_Amplicons | Total_Bases | Covered_Bases | Missed_Bases | Coverage |
| --- | --- | --- | --- | --- | --- | --- | --- | --- | --- |
| 652585 | PPARG | chr3 | 12329343 | 12329524 | 1 | 181 | 177 | 4 | 0.978 |
| 652587 | PPARG | chr3 | 12330430 | 12330638 | 1 | 208 | 208 | 0 | 1 |
| 652584 | PPARG | chr3 | 12353873 | 12353957 | 1 | 84 | 84 | 0 | 1 |
| 652583 | PPARG | chr3 | 12392995 | 12393178 | 1 | 183 | 183 | 0 | 1 |
| 438855 | PPARG | chr3 | 12421197 | 12421435 | 1 | 238 | 238 | 0 | 1 |
| 438856 | PPARG | chr3 | 12422815 | 12422995 | 1 | 180 | 180 | 0 | 1 |
| 438853 | PPARG | chr3 | 12434107 | 12434256 | 1 | 149 | 149 | 0 | 1 |
| 438850 | PPARG | chr3 | 12447375 | 12447585 | 1 | 210 | 210 | 0 | 1 |
| 438857 | PPARG | chr3 | 12458197 | 12458658 | 2 | 461 | 461 | 0 | 1 |
| 652586 | PPARG | chr3 | 12475391 | 12475860 | 2 | 469 | 469 | 0 | 1 |

**Table S3.** Summary of detected variants by Target-sequencing

| Start | Ref/Alt | samples | Function | Rs number | DNA seq | Amino acid | 1000G | gnomAD | SIFT | PolyPhen | HGMD |
| --- | --- | --- | --- | --- | --- | --- | --- | --- | --- | --- | --- |
| 12353772 | G/A | 1 | intronic | - | - | - | - | - | - | - | - |
| 12353993 | G/A | 189 | intronic | rs13073869 | - | - | 0.2646 | - | - | - | - |
| 12392935 | C/G | 1 | upstream | - | - | - | - | - | - | - | - |
| 12393125 | C/G | 41 | nonsynonymous | rs1801282 | c.C155G | p.P12A | 0.0703 | 0.1112 | T | B | - |
| 12393229 | A/G | 1 | intronic | - | - | - | - | - | - | - | - |
| 12421404 | A/G | 1 | nonsynonymous | - | c.A405G | p.Y95C | - | - | D | P | - |
| 12434232 | A/G | 1 | synonymous | rs761941163 | c.A721G | - | - | 0.00001221 | - | - | - |
| 12447627 | T/C | 1 | intronic | rs769946126 | - | - | - | 0.0000139 | - | - | - |
| 12458274 | C/G | 24 | synonymous | rs13306747 | c.C1012G | - | 0.0074 | 0.00406 | - | - | - |
| 12475557 | C/T | 128 | synonymous | rs3856806 | c.C1552T | - | 0.1266 | 0.1348 | - | - | - |
| 12475632 | G/A | 1 | synonymous | rs770898588 | c.G1627A | - | - | 0.00002037 | - | - | - |
| 12422996 | T/C | 1 | intronic | - | - | - | - | - | - | - | - |
| 12475418 | T/C | 1 | nonsynonymous | - | c.T1208C | p.V403A | - | - | D | D | - |
| 12422852 | T/A | 1 | synonymous | - | c.T258A |  | - | - | - |  | - |
| 12421466 | A/G | 1 | intronic | - | - | - | - | - | - | - | - |
| 12421292 | C/T | 1 | nonsynonymous | - | c.C88T | p.H30Y | - | - | D | D | - |
| 12421178 | -/T | 1 | intronic | - | - | - | - | - | - | - | - |

1000G (1000 Genomes Project; National Heart); ExAC_ALL(Exome Aggregation Consortium); ExAC_EAS(Exome Aggregation Consortium East Asia); SIFT (D: deleterious, T: tolerated); PolyPhen-2 (i.e., PPT2) (D: probably damaging, P: possibly damaging, B: benign).

**Table S****4.** Hardy-Weinberg equilibrium

| SNPs | Genotype | Cases (N=233) | | | | Controls (N=93) | | | |
| --- | --- | --- | --- | --- | --- | --- | --- | --- | --- |
|  |  | Actual | Expected | χ2 | P | Actual | Expected | χ2 | P |
| rs1801282 | CC | 211 | 211.52 | 0.57 | 0.75 | 74 | 74.97 | 1.20 | 0.55 |
|  | CG | 22 | 20.96 |  |  | 19 | 17.06 |  |  |
|  | GG | 0 | 0.52 |  |  | 0 | 0.97 |  |  |
| rs3856806 | CC | 147 | 150.08 | 1.62 | 0.44 | 51 | 52.69 | 0.88 | 0.64 |
|  | CT | 80 | 73.84 |  |  | 38 | 34.62 |  |  |
|  | TT | 6 | 9.08 |  |  | 4 | 5.69 |  |  |
| rs13073869 | GG | 96 | 101.79 | 2.86 | 0.23 | 41 | 37.43 | 2.55 | 0.28 |
|  | GA | 116 | 104.43 |  |  | 36 | 43.14 |  |  |
|  | AA | 21 | 26.79 |  |  | 16 | 12.43 |  |  |

Results are expressed as n. Differences between groups were analyzed byχ^2^ text or Fisher’s exact test. **Abbreviations:** SNPs, Single nucleotide polymorphisms

**Table S5.** Multivariate logistic regression analyses of rc with the risk of obesity.

|  | Genotype | unadjusted | | Model 1 | | Model 2 | |
| --- | --- | --- | --- | --- | --- | --- | --- |
|  |  | OR (95CI) | p-value | OR (95CI) | p-value | OR (95CI) | p-value |
| All subjects | CC | 1 |  | 1 |  | 1 |  |
|  | CG | 1.373  (0.704 – 2.677) | 0.352 | 1.293  (0.653 – 2.562) | 0.461 | 1.204  (0.585 – 2.477) | 0.614 |
| OSA  N=233 | CC | 1 |  | 1 |  | 1 |  |
|  | CG | 1.398  (0.580 – 3.368) | 0.455 | 1.366  (0.545 – 3.419) | 0.506 | 1.373  (0.536 – 3.585) | 0.517 |
| Non-OSA  N=93 | CC | 1 |  | 1 |  | 1 |  |
|  | CG | 3.333  (1.012 – 10.983) | 0.048* | 3.752  (1.006 – 13.991) | 0.049* | 2.668  (0.673 – 10.572) | 0.163 |

Model 1: adjusted for age, sex, Smoker and Drinker

Model 2: adjusted for Model 1 + TG, HDL-C and FBG.

**Abbreviations:** OSA, Obstructive sleep apnea; TG, triglycerides; HDL-C, high-density lipoprotein cholesterol; FPG, fasting plasma glucose

*P<0.05.


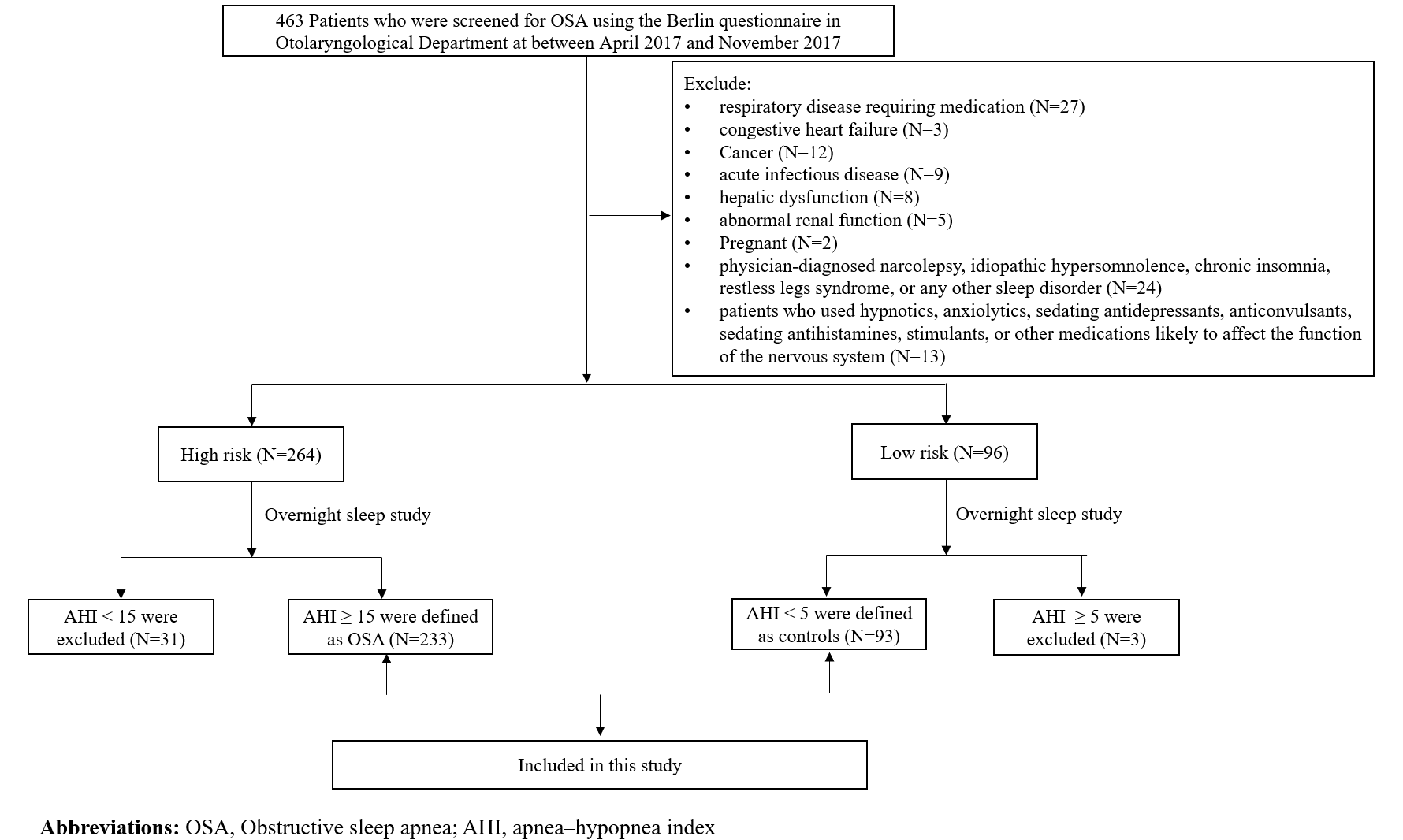
**Figure S1.** Flow chart of inclusion of subjects in this study
